# Detergent-Based Decellularization for Anisotropic Cardiac-Specific Extracellular Matrix Scaffold Generation

**DOI:** 10.3390/biomimetics8070551

**Published:** 2023-11-17

**Authors:** Te-An Chen, Dhavan Sharma, Wenkai Jia, Donggi Ha, Kun Man, Jianhua Zhang, Yong Yang, Yuxiao Zhou, Timothy J. Kamp, Feng Zhao

**Affiliations:** 1 Department of Biomedical Engineering, Texas A&M University, College Station, TX 77843, USA; 2 Department of Mechanical Engineering, Texas A&M University, College Station, TX 77843, USA; 3 Department of Biomedical Engineering, University of North Texas, Denton, TX 76203, USA; 4 Stem Cell and Regenerative Medicine Center, University of Wisconsin-Madison, Madison, WI 53705, USA; 5 Department of Medicine, University of Wisconsin-Madison, Madison, WI 53705, USA

**Keywords:** decellularization, extracellular matrix, cardiac-specific scaffold, cardiac tissue engineering

## Abstract

Cell-derived extracellular matrix (ECM) has become increasingly popular in tissue engineering applications due to its ability to provide tailored signals for desirable cellular responses. Anisotropic cardiac-specific ECM scaffold decellularized from human induced pluripotent stem cell (hiPSC)-derived cardiac fibroblasts (hiPSC-CFs) mimics the native cardiac microenvironment and provides essential biochemical and signaling cues to hiPSC-derived cardiomyocytes (hiPSC-CMs). The objective of this study was to assess the efficacy of two detergent-based decellularization methods: (1) a combination of ethylenediaminetetraacetic acid and sodium dodecyl sulfate (EDTA + SDS) and (2) a combination of sodium deoxycholate and deoxyribonuclease (SD + DNase), in preserving the composition and bioactive substances within the aligned ECM scaffold while maximumly removing cellular components. The decellularization effects were evaluated by characterizing the ECM morphology, quantifying key structural biomacromolecules, and measuring preserved growth factors. Results showed that both treatments met the standard of cell removal (less than 50 ng/mg ECM dry weight) and substantially preserved major ECM biomacromolecules and growth factors. The EDTA + SDS treatment was more time-efficient and has been determined to be a more efficient method for generating an anisotropic ECM scaffold from aligned hiPSC-CFs. Moreover, this cardiac-specific ECM has demonstrated effectiveness in supporting the alignment of hiPSC-CMs and their expression of mature structural and functional proteins in in vitro cultures, which is crucial for cardiac tissue engineering.

## 1. Introduction

Tissue engineering scaffolds provide physical support to incorporated cells, ensuring cell retention and survival at the site of implantation [1]. A tissue-engineered cardiac patch, which functions as a delivery vehicle of human induced pluripotent stem cells (hiPSCs)-derived cardiomyocytes (hiPSC-CMs), represents a promising intervention for rebuilding damaged cardiac tissues. It prevents cell loss in traditional injection treatment, leading to better clinical outcomes [2,3]. Fibrin-based hydrogels have been used to engineer cardiac patch to retain implanted cardiomyocytes in the infarct region, yet they lack cardiac-specific biochemical factors and anisotropic architecture of native myocardium [4,5,6,7]. One potential solution is to use natural extracellular matrix (ECM) derived from organs or tissues because ECM offers a complex microenvironment that closely recapitulates the in vivo environment. However, using allogeneic or xenogeneic tissues can cause undesirable host immune response, batch-to-batch variation, and risk of pathogen transfer [8]. Alternatively, human cells can be pre-screened for pathogens and maintained in a pathogen-free environment. Therefore, ECM secreted from human cells has the potential to provide a safer, more reproducible, and personalized microenvironment [9,10].

In natural myocardium, cardiac fibroblasts contribute to ECM deposition and growth factor secretion, which regulate the fate of cardiomyocytes, endothelial cells, and smooth muscle cells, dictating their differentiation, migration, proliferation, and overall homeostasis [11,12]. In addition, the myocardium exhibits high degree of alignment in its native cellular and ECM organization [13,14]. Thus, decellularized ECM from aligned cardiac fibroblast cell sheets holds great potential to support hiPSC-CMs by providing a microenvironment rich in cardiac-specific biochemical and signaling cues, as well as an anisotropic template for cellular alignment. Moreover, hiPSC-derived cardiac fibroblasts (hiPSC-CFs) exhibit better sustainability in proliferative capacity compared to human primary cardiac fibroblasts [15], making them a good candidate for the fabrication of cell-derived ECM.

To ensure adequate decellularization of tissue, complete removal of cellular and nuclear components of the host cells is necessary to minimize immunological response. Preserving structural ECM proteins and other biomacromolecules is also critical to maintain ECM function [16]. Decellularization ECM has been widely applied to tissues and organs reconstruction, including corneal [17,18,19], musculoskeletal [20,21,22], cardiac [23,24,25], and neural tissues [26,27,28]. Additionally, various decellularization methods have been employed to generate decellularized ECM from cell sheets [17,29,30,31], tissues [32,33,34,35], and organs [36,37,38,39,40]. Current methods of decellularization include chemical, enzymatic, and physical treatments [41,42]. Chemical treatments, such as ionic, non-ionic, or zwitterionic detergents, can solubilize cell membranes and dissociate DNA from proteins to effectively remove cellular components. However, ionic detergents are powerful and tend to disrupt ECM proteins more than other detergents [31,43]. Enzymatic treatment, such as nuclease or protease, can selectively target and eliminate cellular components. Nuclease removes nucleic acids while protease cleaves proteins to separate undesirable cellular components from ECM. However, prolonged exposure of enzymes can cause loss of glycosaminoglycan (GAG), laminin, and collagen IV, which comprises mechanical stability of ECM [44]. Physical treatment, such as freeze–thaw cycle, agitation, perfusion, uses osmotic or mechanical force to disintegrate cellular components. Precise control of the force applied is required to prevent tissue damage [31,41,45,46]. Therefore, the choice of decellularization method depends on the specific requirements of the application and the type of tissue to be decellularized. Because each method has its own advantages and disadvantages, a combination of multiple methods is often necessary to achieve complete decellularization [6,47].

The objective of the current study was to create an anisotropic cardiac-specific ECM scaffold via decellularizing aligned hiPSC-CF cell sheets. Two detergent-based decellularization methods were compared: (1) a combination of ethylenediaminetetraacetic acid and sodium dodecyl sulfate (EDTA + SDS), and (2) a combination of sodium deoxycholate and deoxyribonuclease (SD + DNase). Morphological analysis of ECM-specific fibrous proteins and quantification of ECM-specific key structural biomacromolecules, ECM-bound growth factors, and residual DNA concentration were used to evaluate the effectiveness of each method. Additionally, the effectiveness of hiPSC-CF-derived ECM (hiPSC-CF-ECM) in supporting the alignment and functional maturation of hiPSC-CMs was evaluated by determining the specific structural proteins expression of cardiomyocytes grown on the decellularized anisotropic ECM architecture. Overall, the current work delineates an effective decellularization method to create an aligned cardiac-specific ECM for cardiac tissue engineering.

## 2. Materials and Methods

### 2.1. hiPSC-CFs Differentiation and Culture

hiPSCs (DF19-9-11T, WiCell Research Institute Inc., Madison, WI, USA) maintained in StemFlex media (Thermo Scientific, Waltham, MA, USA) on Matrigel (GFR, Corning Inc., Corning, NY, USA)-coated plates were dissociated using Versene solution (Gibco, Thermo Scientific, Waltham, MA, USA) and seeded on Matrigel-coated plates at a density of 200,000 cells/cm^2^ in mTeSR1 medium (STEMCELL Technologies, Vancouver, BC, Canada) containing 10 µM ROCK inhibitor (Y-27632, Tocris, Bristol, UK). Cells were cultured in mTeSR1 medium for 5 days with medium change every day until fully confluent (day 0). At day 0, cells were treated with 12 µM CHIR99021 (Tocris) in RPMI/B27-insulin media (B27 Supplement minus insulin, Gibco) for 24 h. At day 1, the medium was changed to RPMI/B27-insulin and cells were cultured in the medium for 42 h (day 2.75). At day 2.75, the medium was changed to CFBM [15] supplemented with 75 ng/mL bFGF (WiCell Research Institute). Cells were fed with CFBM plus 75 ng/mL bFGF every other day until day 20 when they were harvested for flow cytometry to assess the purity of the differentiated hiPSC-CFs and passaged. Pure hiPSC-CFs (>70% TE7^+^) were cryopreserved and used for decellularization.

### 2.2. Fabrication of Anisotropic Cardiac Fibroblast Cell Sheets

The nano-grated polydimethylsiloxane (PDMS) substrates used in this study were replicated from a nanostructured master mold that was fabricated using electron beam lithography. To create the substrates, a mixture of PDMS resin and curing agent (Slygard 184 Silicone Elastomer Kit; Dow Corning, Midland, MI, USA) was casted onto the master mold and cured at 70 °C for 4 h [48]. Prior to cell culture, the PDMS substrates were coated with polydopamine and collagen I as described in the previous publication [49]. Briefly, PDMS were immersed in 0.01% *w*/*v* 3-hydroxytyramine hydrochloride (Dopamine-HCl) (ACROS Organics, Fisher Scientific, Hampton, NH, USA) for 24 h followed by ethylene oxide sterilization. Polydopamine-coated PDMS were immersed in bovine collagen (20 μg/mL) (Sigma Aldrich, St. Louis, MO, USA) for 2 h before cell seeding [49]. Passage 4–6 of hiPSC-CFs was seeded on 24-well size nano-grated PDMS for 2 weeks at a density of 10,000 cells/cm^2^. The cells were cultured in FibroGro Complete Media Kit (Millipore SCMF001, by replacing Glutamine with the same amount of GlutaMAX (Thermo Scientific)), supplemented with 10 mL fetal bovine serum (FBS, R&D systems, Minneapolis, MN, USA). The hiPSC-CF cell sheets were developed after 2 weeks of seeding by changing culture medium at every 48 h.

### 2.3. Decellularization Methods to Fabricate hiPSC-CF-ECM

Removal of cellular and nuclear components of the hiPSC-CF sheets were achieved by two different decellularization methods. In the first decellularization (EDTA + SDS) method, hiPSC-CF sheets were immersed into decellularization solution A, containing 1 M NaCl, 10 mM Tris (Bio-Rad, Hercules, CA, USA), and 5 mM EDTA (Sigma) and incubated for 1 h at room temperature (RT) on slow shaker. Next, sheets were thoroughly rinsed with phosphate buffered saline (PBS) and immersed into decellularization solution B, containing 0.1% SDS (Sigma), 10 mM Tris, and 25 mM EDTA and incubated at RT for 30 min on slow shaker followed by rinsing via PBS. Sheets were then immersed in Dulbecco’s Modified Eagle Medium (DMEM, Fisher Scientific) with 20% FBS for 48 h at RT on slow shaker. Finally, decellularized ECM derived from hiPSC-CF sheets were thoroughly rinsed with PBS and stored at −80 °C until further use.

In the second decellularization (SD + DNase) method, hiPSC-CF sheets were immersed in a decellularization solution containing 0.25% Triton X-100 (Fisher Scientific) and 0.25% SD (Sigma) prepared in PBS and incubated at 37 °C for 24 h on slow shaker. Next, sheets were thoroughly washed with PBS and immersed in DMEM medium supplemented with 20% FBS at 4 °C on slow shaker for 48 h, followed by another PBS rinse. After rinse, sheets were treated with 100 mg/mL RNase (Roche Diagnostics, Indianapolis, IN, USA) and 150 IU/mL DNase (Sigma) solution prepared in PBS containing 50 mmol/L MgCl_2_ for 24 h at 37 °C. Finally, decellularized ECM derived from hiPSC-CF sheets were thoroughly rinsed with PBS and can be stored at −80 °C until further use. Decellularized ECMs were sterilized by immersing in 70% ethanol under UV for 30 min, followed by 3 washes of sterile PBS. Prior to hiPSC-CMs seeding, ECMs were immersed in cardiomyocyte-specific cell culture media and were incubated overnight at 37 °C and 5% CO_2_.

### 2.4. Morphological Analysis of ECM Key Structural Proteins

hiPSC-CF-ECM with a diameter of 14 mm was fixed with 4% paraformaldehyde (Fisher Scientific) for 30 min and then blocked with 1% bovine serum albumin (BSA, Sigma) in a 0.2% Triton X-100 (Fisher Scientific) solution for 30 min. The samples were stained with primary antibodies targeting collagen I, collagen IV, fibronectin, and laminin (Abcam, Cambridge, MA, USA) for overnight at 4 °C. Next, the sample were stained with goat-anti mouse Alexa Fluor^TM^ 488 conjugated or Alexa Fluor^TM^ 594 conjugated secondary antibodies (Invitrogen, Fisher Scientific) for 1 h at RT. Cell nuclei were stained with 4′,6-diamidino-2-phenylindole (DAPI, Sigma) for 5 min at RT. Z-stacking in confocal microscopy (Olympus FV-1000, Tokyo, Japan) was performed to image ECM proteins and measure ECM thickness. Six non-overlapping images were captured from each biological triplicate (*n* = 3) in each treatment group. Nanofibrous architecture of ECM scaffolds were observed by field emission scanning electron microscopy (FE-SEM). Decellularized ECM samples were fixed with 2% glutaraldehyde for 15 min and then were washed with PBS for 3 times. Next, the samples were treated with a graded series of ethanol (50%, 70%, 95% and 100% ethanol) for dehydration. Finally, samples were then dried in Hexamethyldisilane (Sigma) and imaged with Hitachi S-4700 field emission scanning electron microscope (Tokyo, Japan).

### 2.5. ECM Components Characterization

The quantification of structural components and growth factors embedded in the hiPSC-CF-ECM were performed by enzyme-linked immunosorbent assay (ELISA) using commercially available kits following manufacturer’s instructions. hiPSC-CF-ECM cultured in a 24-well plate with a diameter of 14 mm were used for all quantifications. Structural components, including soluble and insoluble collagen, fibronectin, elastin, and GAG, were quantified from the cell sheets before decellularization and the ECM after two decellularized methods. The protein amount was reported as the ratio of the amount after treatments to the amount before treatments. Soluble and insoluble collagen were quantified by using Sircol Assay (Biocolor Ltd., Carrickfergus, UK). 0.5 M acetic acid was used to extract soluble collagen while the non-dissolved ECM residue was treated with fragmentation reagent. The extracted collagen was measured following the manufacturer’s protocol. ECM-bound sulfated GAG content was determined by Blyscan Sulfated Glycosaminoglycan Assay Kit (Biocolor Ltd., Carrickfergus, UK). Samples were digested using papain extraction reagent (0.1 mg/mL papain) and heated at 65 °C for 3 h. After digestion, samples were centrifuged at 10,000× *g* for 10 min. Supernatant was collected and assayed following the manufacturer’s protocol. Elastin was determined by the Fasting Elastin Assay Kit (Biocolor Ltd.). Samples were treated with 0.25 M oxalic acid at 100 °C for 1 h to convert insoluble elastin to water soluble α-elastin, then centrifuged at 13,000× *g* for 10 min afterwards. Supernatant was collected and assayed following the manufacturer’s protocol. Fibronectin was quantified by ELISA (R&D Systems). Absorbance was measured using a microplate reader (Cytation 5, Biotek, Winooski, VT, USA) and was used to calculate the protein amount. Similarly, growth factors such as basic fibroblast growth factor (bFGF), vascular endothelial growth factor (VEGF), insulin-like growth factor (IGF), angiotensin II, and endothelin I were also determined by ELISA kits (R&D Systems). Growth factors in cellular components were extracted as previously described [10]. Briefly, growth factors of ECM scaffold were reconstituted in collecting buffer by using 1 mL of extraction buffer and sonication. Samples were shaken on an orbital shaker at 4 °C overnight. Growth factor concentration was then determined following the manufacturer’s protocol. Absorbance was measured by a microplate reader (Cytation 5, Biotek) and used to calculate the protein amount.

### 2.6. hiPSC-CMs Differentiation and Culture

hiPSCs (DF19-9-11T, WiCell Research Institute) were cultured and maintained as described under hiPSC-CFs differentiation and culture section. The hiPSC-CMs were differentiated using the GiWi protocol [50]. In brief, hiPSCs were dissociated using Versene solution (Gibco) and seeded on Matrigel (GFR, Corning)-coated plates at a density of 200,000 cells/cm^2^ in mTeSR1 (STEMCELL Technologies) medium containing 10 µM ROCK inhibitor (Y-27632, Tocris). Cells were cultured in mTeSR1 medium for 5 days with medium change every day until fully confluent (day 0). At day 0, cells were treated with 12 µM CHIR99021 (Tocris) in RPMI/B27-insulin media (B27 Supplement minus insulin, Gibco) for 24 h. At day 1, the medium was changed to RPMI/B27-insulin and cells were cultured in the medium for 48 h (day 3). At day 3, a combined medium was prepared by collecting half of the old medium from each well and mixing with same volume of fresh RPMI + B27 without insulin medium and supplemented with 5 µM IWP2 (Tocris), and cells were treated with IWP2 in the medium for 48 h (day 5). At day 5, the medium was changed to RPMI + B27 without insulin and cells were cultured for 2 days. On day 7 the medium was changed to RPMI + B27 with insulin (B27 supplement, Gibco), and the cells were fed every other day until day 15 when they were harvested for flow cytometry to assess the purity of the differentiated hiPSC-CMs and cryopreservation.

Greater than 70% purity (% of cTnT^+^ cells) of the hiPSC-CMs were thawed and plated on Synthemax (Corning)-coated plates in the EB20 media [51]. After 2–3 days when hiPSC-CMs were attached, the media was changed to RPMI + B27 inulin (Gibco) and cells were maintained in the media for a total of 7 days when contracting cells resumed. After 7 days, the hiPSC-CMs were dissociated with TrypLE 10X (Gibco) and seeded onto the hiPSC-CF-ECM scaffold for an additional 7 days until further characterizations.

### 2.7. hiPSC-CMs Immunofluorescence Staining and Imaging

After 7 days’ culture, immunofluorescence staining was performed to determine the presence of native cardiomyocyte-specific key structural and functional proteins. hiPSC-CMs cultured on hiPSC-CF-ECM were fixed with 4% paraformaldehyde (Fisher Scientific) for 30 min and blocked with 1% BSA prepared in a 0.2% Triton X-100 solution for 30 min. The samples were stained with primary antibodies for overnight at 4 °C targeting the gap junction protein connexin 43, sarcomeric alpha-actinin, cardiac troponin T, and F-actin. Followed by overnight primary antibody incubation, samples were thoroughly washed with blocking buffer and stained with goat-anti mouse Alexa Fluor^TM^ 488 conjugate or Alexa Fluor^TM^ 594 conjugated secondary antibodies (Invitrogen, Fisher Scientific) for 1 h at RT. Cell nuclei were stained with 4′,6-diamidino-2-phenylindole (DAPI, Sigma) for 5 min at RT. hiPSC-CMs were imaged with the Olympus FV-1000 confocal microscope.

### 2.8. hiPSC-CMs Beating Analysis

The beating rate and the maximum principal strain variation were calculated from each time-lapse video using Digital Image Correlation (DIC) in the DaVis D10.2 software (LaVison, Gottingen, Germany). DIC is a non-contact optical technique that can track the motion and shape change in a material by analyzing consecutive deformed images collected over time. During DIC analysis, each image was divided into smaller regions (subset) and the displacement of each subset was tracked individually. The result of DIC is a 2D displacement vector field. The 2D maximum principal strain field, which characterizes the stretch of material during a beating cycle, is calculated by measuring the gradient of the displacement field. In this study, videos containing approximately 400 images (704 µm × 532 µm) were analyzed with a subset size of dimension ~94 µm and a step size of 3.5 µm (overlap between consecutive subsets). The beating rate is determined by analyzing the number of peaks from the periodic variation in the maximum principal strain in ROI over time. Three regions of interest with high cell density were selected from each video, and the subset with the maximum deformation in each region was chosen for further analysis.

### 2.9. Statistical Analysis

Statistical comparisons between experimental groups were performed by one-way ANOVA and Tukey’s post hoc test using GraphPad Prism 9 software (GraphPad Software, Boston, MA, USA). All experiments were performed in biological triplicates with technical quadruplicates. For image-based analysis, six non-overlapping images were captured from each biological triplicate (*n* = 3) in each experimental group and analyzed. Results are displayed as the mean ± standard deviation and were considered statistically significant for * *p* < 0.05, ** *p* < 0.01, *** *p* < 0.001, **** *p* < 0.0001.

## 3. Results

### 3.1. Characterization of ECM before and after Decellularization

**Figure 1:**
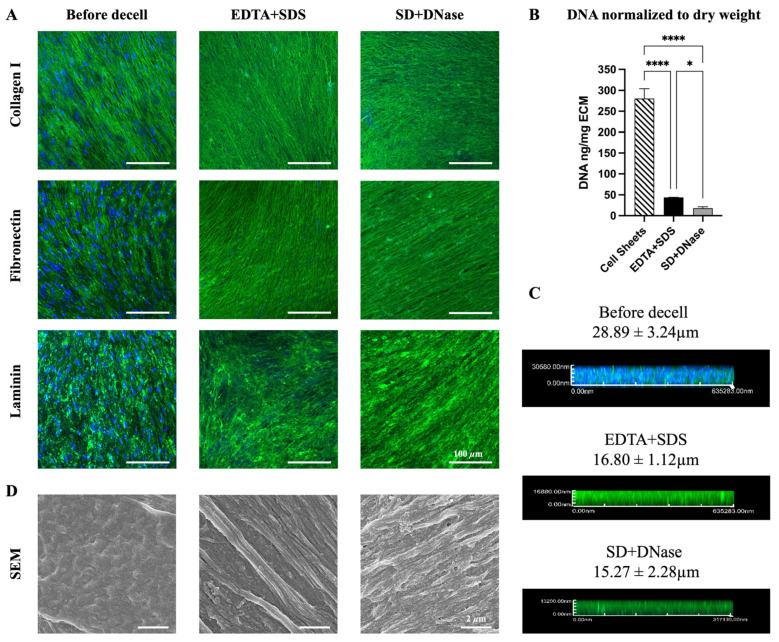
Morphological characteristics of hiPSC-CF sheets before and after decellularization. (**A**) Immunofluorescence images of hiPSC-CF sheets and ECM after two decellularization processes reveal anisotropic organization of key ECM proteins, collagen I (green), fibronectin (green), laminin (green), and cell nuclei via DAPI (blue). (**B**) Quantification of DNA concentration in hiPSC-CF sheets and hiPSC-CF-ECM followed by EDTA + SDS and SD + DNase treatments. (**C**) Measurement of hiPSC-CF sheets thickness before and after decellularization via Z-stacking in confocal imaging. (**D**) Morphological assessment of hiPSC-CF sheets and nanofibrous ECM bundles in hiPSC-CF-ECM via FE-SEM. Results displayed as the mean ± standard deviation and were considered statistically significant for * *p* < 0.05, **** *p* < 0.0001.

To obtain an anisotropic ECM-fiber architecture, hiPSC-CFs were cultured on nano-gated PDMS substrates for two weeks to form aligned hiPSC-CF sheets. The PDMS nano-grooves were equally spaced at 450 nm width and 300 nm depth. After the two-week culture, the hiPSC-CF sheets were decellularized using either EDTA + SDS or SD + DNase treatment to produce aligned nanofibrous ECM scaffolds. Both the hiPSC-CF sheets and ECMs displayed anisotropic organization of key ECM-specific proteins, including collagen I, fibronectin, and laminin, as evidenced by immunofluorescence staining (Figure 1A and Supplementary Figure S1). Following decellularization, the preservation of these proteins was maintained, and their density increased due to the removal of cellular components in both EDTA + SDS and SD + DNase groups. Unlike the cell sheets, the ECMs produced by both methods showed undetectable DAPI signals, indicating efficient removal of nucleic acids (Figure 1A). The decellularization efficacy for both methods was quantified by measuring DNA concentration via pico-green assay. As shown in Figure 1B, the SD + DNase treatment was more efficient at removing double-stranded DNA from ECM compared to the EDTA + SDS treatment. The remaining DNA concentration after SD + DNase treatment (18.0 ± 4.3 ng/mg of ECM) was significantly lower (*p* < 0.05) than that of the EDTA + SDS treatment (43.8 ± 0.62 ng/mg of ECM), and both treatments showed significantly lower (*p* < 0.0001) DNA concentration compared with the cell sheets. (Figure 1B). Z-stack confocal images revealed that the removal of cellular components from cell sheets resulted in a reduction in the ECM thickness. Although the EDTA + SDS treatment preserved slightly thicker ECM (16.80 ± 1.12 μm) than the SD + DNase treatment (15.27 ± 2.28 μm), this difference was not statistically significant (Figure 1C). High resolution images obtained from FE-SEM showed that nanoscale ECM fibers organized in the anisotropic orientation (Figure 1D). It was observed that the fibrous structure was better exposed when the cell sheets were decellularized with EDTA + SDS treatment.

### 3.2. Quantification of Major ECM Composition

**Figure 2:**
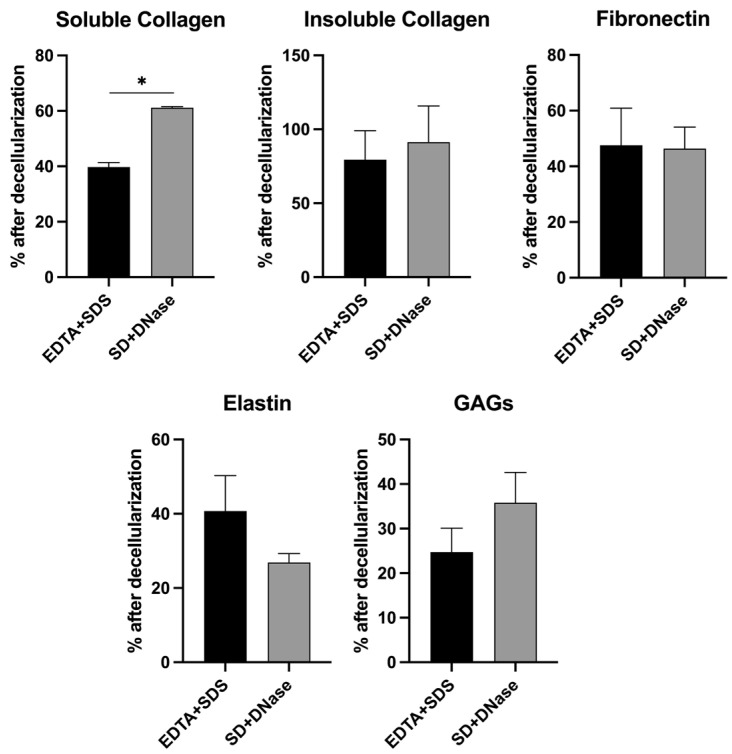
Quantification of major ECM macromolecules in hiPSC-CF-ECM. Comparison of soluble collagen, insoluble collagen, fibronectin, elastin, and GAG in hiPSC-CF-ECM after decellularization using the EDTA + SDS and SD + DNase methods. Results were presented as the mean ± standard deviation and were considered statistically significant for * *p* < 0.05.

To determine the effect of decellularization methods on the composition of the ECM, major ECM macromolecules including soluble collagen, insoluble collagen, fibronectin, elastin, and GAG were quantified (Figure 2). The results showed significant differences in the levels of soluble collagen between the EDTA + SDS and SD + DNase treatment groups (39.79 ± 5.35% in EDTA + SDS versus 54.46 ± 6.30% in SD + DNase, *p* < 0.05), with the SD + DNase treatment group preserving higher levels of soluble collagen. However, there were no significant differences in the levels of insoluble collagen (79.48 ± 19.53% in EDTA + SDS versus 91.32 ± 24.48% in SD + DNase) and fibronectin (47.55 ± 12.32% in EDTA + SDS versus 46.34 ± 7.78% in SD + DNase). The level of elastin after decellularization showed a non-significantly higher level in EDTA + SDS treatment group compared with SD + DNase treatment group (40.72 ± 9.57% in EDTA + SDS versus 26.85 ± 2.41% in SD + DNase). Conversely, the level of GAG after decellularization was observed to be non-significantly higher in the SD + DNase treatment group compared to the EDTA + SDS treatment group (24.45 ± 5.36% in EDTA + SDS versus 35.80 ± 6.80% in SD + DNase) (Figure 2).

### 3.3. Quantification of Major ECM Growth Factors

**Figure 3:**
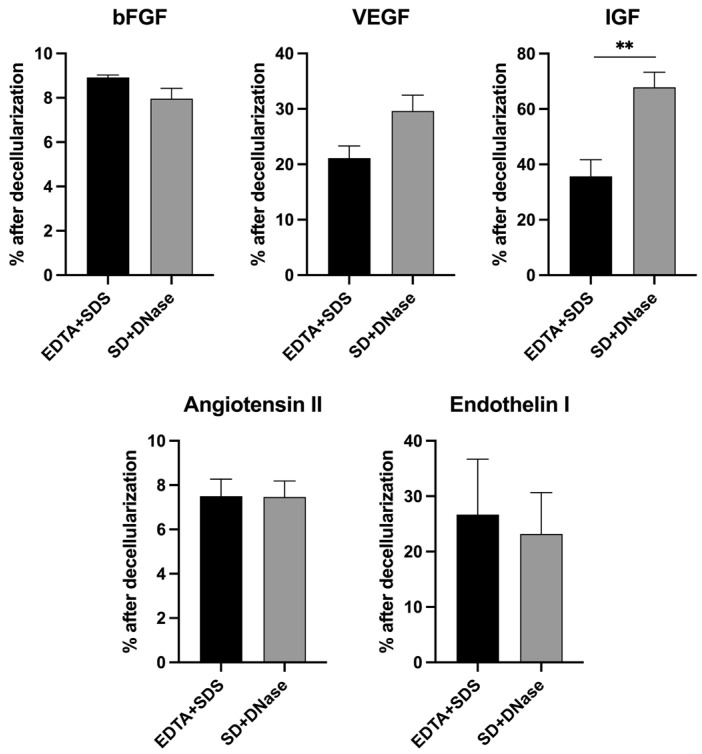
Quantification of ECM-embedded growth factors in hiPSC-CF-ECM. Comparison of bFGF, VEGF, IGF, angiotensin II, and endothelin I in hiPSC-CF-ECM after decellularization using the EDTA + SDS and SD + DNase methods. Results were presented as the mean ± standard deviation and were considered statistically significant for ** *p* < 0.01.

The growth factors embedded within the ECM after decellularization were quantified using ELISA. The levels of IGF exhibited a significant difference (35.67 ± 6.02% in EDTA + SDS versus 67.85 ± 5.46% in SD + DNase, *p* < 0.01) between the two decellularization treatments, with the SD + DNase treatment group showing significantly higher levels of IGF preserved compared to the EDTA + SDS treatment group. However, there were no significant differences observed for the levels of bFGF (8.92 ± 0.11% versus 7.76± 0.46%), angiotensin II (7.50 ± 0.77% versus 7.47 ± 0.72%), and endothelin I (26.67 ± 10.02% versus 23.19 ± 7.75%) between the EDTA + SDS and SD + DNase treatment groups. The levels of VEGF preserved post decellularization were moderately higher in the SD + DNase treatment group compared to the EDTA + SDS treatment group, although this difference was not significant (21.12 ± 2.20% in EDTA + SDS versus 29.62 ± 2.87% in SD + DNase) (Figure 3).

### 3.4. The Structural and Functional Maturation of hiPSC-CMs on ECM Scaffold

**Figure 4:**
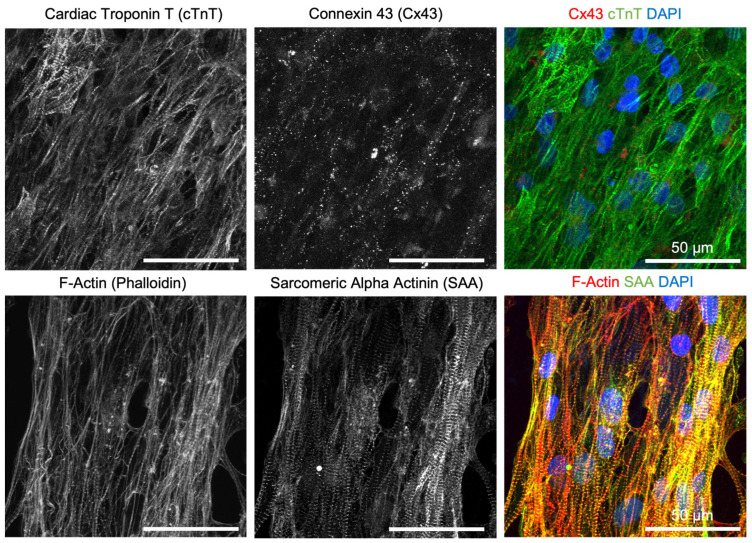
Evaluation of native cardiomyocyte-specific structural and function protein organization in hiPSC-CMs cultured on hiPSC-CF-ECM. hiPSC-CMs cultured on hiPSC-CF-ECM were oriented in aligned direction following ECM fiber anisotropy. hiPSC-CMs showed mature organization of key functional proteins including cardiac troponin T (cTnT, green), gap junction proteins connexin 43 (Cx43, red) and structural proteins sarcomeric alpha-actinin (SAA, green), and F-actin (Phalloidin, red).

To evaluate the ability of decellularized scaffolds to support the in vitro cell culture, hiPSC-CMs were cultured on the hiPSC-CF-ECM decellularized with EDTA + SDS method for 7 days, followed by examination of cell morphology and function. hiPSC-CMs cultured on the hiPSC-CF-ECM organized into a compact and highly aligned cell layer (Figure 4 and Supplementary Figure S2), following the anisotropic direction of ECM nanofibers, resembling the structure of native myocardium. The cells also exhibited expression and organization of key structural proteins, including sarcomeric alpha-actinin and F-actin, indicating matured sarcomere structure (Figure 4). Furthermore, hiPSC-CMs displayed matured expression of native cardiomyocyte-specific functional proteins such as gap junction protein connexin 43 and cardiac troponin T.

### 3.5. Beating Analysis of hiPSC-CMs on ECM Scaffold

The contractile properties of hiPSC-CMs cultured on hiPSC-CF-ECM scaffolds were analyzed. This involved measuring two key parameters: the maximum principal strain during beating, and duration of the beating cycles. The onset of spontaneous beating of hiPSC-CMs was observed typically around day 5 post seeding. The beating of cardiomyocytes was video-recorded for subsequent analysis (Supplementary Videos S1–S3). The maximum principal strain variation (0.005 ± 0.004) and duration of the beating cycles (3.26 ± 0.16 s/beat) of hiPSC-CMs on ECM scaffolds were analyzed. These findings provided insight into the contractile capacity of hiPSC-CMs (Supplementary Figure S3).

## 4. Discussion

In vitro generation of cell-derived ECM from various fibroblast types enables customization of biological properties to better match specific tissue applications [52,53,54]. Compared to synthetic materials, ECM contains an abundance of tissue-specific protein fractions and bioactive molecules, leading to a compositional complexity that closely resembles that of the native microenvironment [10,55,56]. As such, the ECM scaffold obtained from hiPSC-CF cell sheets is expected to contain structural proteins and growth factors that are specific to the cardiac microenvironment, making it a promising substrate for the maturation of hiPSC-CMs and for use in cardiac patch engineering. To obtain cardiac-specific ECM, an effective decellularization method is required, which involves removing cellular components while maximally preserving the ECM structure as well as ECM-bound growth factors. The efficacy of a successful decellularization method can be evaluated based on key metrics such as the absence of DAPI signal and requiring less than 50 ng of residual DNA per mg of ECM dry weight [57,58].

To obtain a cardiac-specific ECM scaffold, in this study, we evaluated two detergent-based decellularization methods. The first method involved a combination of EDTA + SDS, while the second method utilized a combination of SD + DNase. EDTA is a chelator that binds to metallic ions to disrupt cell adhesion to ECM. DNase is a nuclease that hydrolyzes phosphodiester bonds in DNA to eliminate residual nucleic acids. SDS and SD are ionic detergents that can solubilize cytoplasmic and nucleic membranes and dissociate DNA from protein through protein denaturation [41,57]. Confirmed by immunofluorescence staining and DNA assay, the utilization of combinations of EDTA + SDS and SD + DNase has both achieved the standard of successful decellularization (Figure 1A–C). In addition, the thickness of decellularized ECM is a critical factor in cardiac patch engineering. A scaffold that is too thin fails to provide adequate mechanical support during patch preparation, while an overly thick scaffold can hinder the penetration of cells into the scaffold. The thickness of our hiPSC-CF-ECM scaffold, fabricated after 2 weeks of culture, is sufficient to support cardiomyocyte culture and contraction (Figure 4 and Supplementary Figure S3), while eliminating the necessity of cell penetration. Moreover, the FE-SEM images revealed that the SDS treatment better exposed the ECM’s fibrous protein structures (Figure 1D), which could be attributed to the stronger ability of SDS to break non-covalent bonds in proteins through electrostatic interactions, thereby unwinding the protein fibers [59,60].

To maintain the functionality of the decellularized tissue scaffold, it is crucial to preserve the composition and architecture of the ECM. The native cardiac ECM is a nanofibrous network of fibrillar and non-fibrillar components [61]. Fibrillar components, such as collagens and elastin, provide mechanical support, structural stability, and elasticity to the heart tissue, while non-fibrillar components, such as fibronectin, laminin, and GAG, regulate cell proliferation, migration, differentiation, and adhesion by interacting with cell-surface receptors [61,62]. Thus, it is critical to preserve both the fibrillar and non-fibrillar structure during decellularization. Overall, there was a 40–70% loss of ECM macromolecules during the decellularization process, with the exception of insoluble collagen, which is the main drawback of using ionic detergents [57]. Stronger detergent, such as SDS relative to SD, is more disruptive to ECM proteins, consistent with our protein quantification results (Figure 2). Several literatures also reported ECM protein losses when applying ionic detergents to prepare decellularized ECM [30,31,63]. Although the partial loss of structural proteins is inevitable, the hiPSC-CF-ECM still possessed sufficient protein retention, supported by the seeded cardiomyocytes showing matured expression and organization of structural and functional proteins (Figure 4). Our results indicated that the SD + DNase method better maintained the soluble collagen (Figure 2) consistent with a previous study on decellularization of porcine aortic and blood vessels, which reported higher soluble collagen content in SD treatment groups compared with SDS treated samples [63]. Compared to SDS, SD is a milder detergent that is more efficient in preserving bioactivity and protein structures. A previous study demonstrated that SD caused less disruption and better maintained ECM integrity when treating mouse lung tissues [64]. However, one problem of SD treatment is the occurrence of DNA agglutination on the tissue surface (Figure 1B), which can compromise the efficacy of the decellularization process. Fortunately, the use of DNase can mitigate this problem [60].

ECM-bound growth factors play a vital role in supporting cell viability, maturation, and maintaining scaffold functionality [65]. The native cardiac ECM contains various growth factors such as VEGF, FGF, IGF, angiotensin II, and endothelin I. VEGF is known to regulate angiogenesis by inducing endothelial cell proliferation, migration, tube formation, and survival in the heart [66]. Similarly, FGF also supports angiogenesis [67]. IGF, known as the cardiac growth hormone, is crucial for protecting cardiomyocytes from oxidative stresses and apoptosis [68,69]. Angiotensin II and endothelin I are peptide hormones that are involved in regulating blood pressure, inducing cardiac hypertrophy, and enhancing contractile function and growth of cardiomyocytes [70,71,72]. Our quantitative analysis demonstrated that both decellularization methods were able to effectively preserve a substantial amount of all growth factors, while SD + DNase method showed 1.9 times more IGF than the SDS treated samples (Figure 3).

This study aimed to determine the most appropriate decellularization method by comparing the efficacy of the EDTA + SDS and SD +DNase methods in removing cellular component while preserving structural proteins and growth factors. Both methods met the standard of decellularization. However, this study also took into consideration the processing time required for the decellularization methods, and observed that the EDTA + SDS method required 48 h less than the SD + DNase method, making it a more appealing method for future applications.

To evaluate the efficacy of the cardiac-specific hiPSC-CF-ECM in engineering cardiac patches, hiPSC-CMs were grown on the aligned hiPSC-CF-ECM and their phenotypic expression and maturation were investigated. The results showed that the hiPSC-CMs cultured on hiPSC-CF-ECM exhibited highly aligned sarcomere structures, which are the fundamental contractile units of myofibrils in cardiomyocytes (Figure 4). The structural protein cardiac troponin T regulates the heart contraction by modulating calcium-dependent interaction between actin and myosin [73], while sarcomeric alpha-actinin crosslinks with actin filaments to organize the thin filaments into a repeating pattern, contributing to the contractile strength of the cardiac muscle fibers and playing a crucial role in the mechanical and functional properties of the cardiomyocytes [74]. Additionally, the gap junction protein connexin 43 forms channels between adjacent cells, allowing for the exchange of ions and small molecules and contributing to the propagation of action potentials for synchronous contraction of the heart [75]. The observed organization of these structural and functional proteins, as well as the cardiomyocyte contractility (Supplementary Figure S3), suggest that hiPSC-CF-ECM has the potential to be used in cardia patch engineering.

## 5. Conclusions

This study aimed to create an anisotropic cardiac-specific ECM scaffold from hiPSC-CF cell sheets. Two decellularization methods, EDTA + SDS and SD + DNase, were compared in terms of their effects on the ECM morphology, architecture, composition, and growth factors embedded within the ECM scaffold. The results revealed that the EDTA + SDS treatment effectively removed DNA content while preserving major ECM proteins and growth factors that are essential for desirable cellular responses. Furthermore, this decellularized aligned cardiac-specific ECM scaffold facilitated the matured organization of key cardiomyocyte-specific structural and functional proteins of hiPSC-CMs, indicating its promise for cardiac tissue engineering.

## Data Availability

The data presented in this study are available on request from the corresponding author.

## Supplementary Materials

The following supporting information can be downloaded at: https://www.mdpi.com/article/10.3390/biomimetics8070551/s1, Figure S1: Quantification of alignment of hiPSC-CF sheets and hiPSC-CF-ECM; Figure S2: Quantification of cell alignment of hiPSC-CMs; Figure S3: Evaluation of hiPSC-CMs contractility on ECM scaffold; Videos S1–S3: Beating videos of hiPSC-CM on hiPSC-CF-ECM.
