# Supplementary material for: Detergent-Based Decellularization for Anisotropic Cardiac-Specific Extracellular Matrix Scaffold Generation
Source: Biomimetics (Basel). 2023 Nov 17;8(7):551. doi: 10.3390/biomimetics8070551 (PMC10669368; doi:10.3390/biomimetics8070551)
Supplement: Supplementary file 1 [file biomimetics-08-00551-s001.zip › Supplementary Data.pdf]

Supplementary data

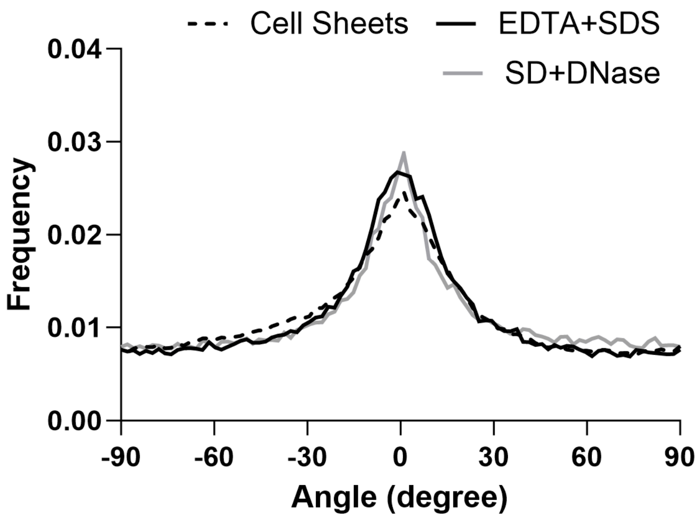

Figure S1. Quantification of alignment of hiPSC-CF sheets and hiPSC-CF-ECM. The cell alignment was quantified by measuring the angle of collagen-I fiber stained in cell sheets (before decell) and ECM decellularized by EDTA + SDS and SD + DNase methods. This suggested that the ECM secreted by hiPSC-CFs was organized by underlined aligned pattern.

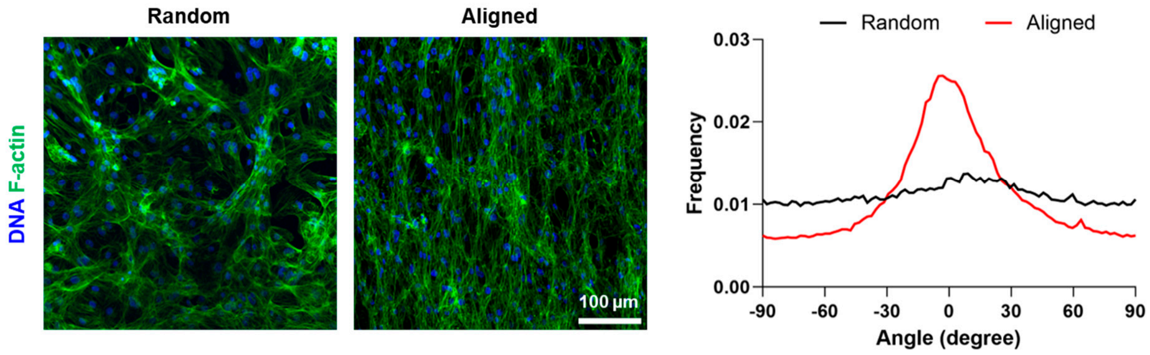

Figure S2. Quantification of cell alignment of hiPSC-CMs cultured on glass substrate (random pattern) and aligned hiPSC-CF-ECM (aligned pattern). The cell alignment was quantified by measuring the angle of F-actin stained in hiPSC-CMs. The cardiomyocytes on the aligned hiPSC-CF-ECM showed a narrower distribution of F-actin angles, suggesting that the patterned ECM guided the cardiomyocytes and made them more aligned.

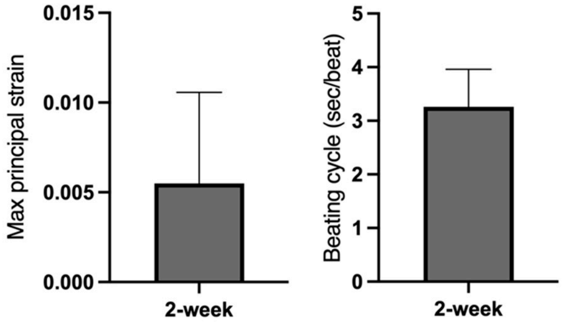

Replicate	Area	Max Principal Strain (x 10^{-3})	Beating Cycle (beat/sec)
1	1	5.02	2.39
	2	1.29	2.36
	3	3.03	2.39
2	1	4.80	3.97
	2	1.61	3.97
	3	4.97	3.97
3	1	1.15	3.45
	2	11.58	3.45
	3	16.00	3.42

Figure S3. Evaluation of hiPSC-CMs contractility on ECM scaffold. Maximum principal strain and beating cycle duration of cardiomyocyte showed potential functional maturation of hiPSC-CMs on hiPSC-CF-ECM. Results displayed as mean ± standard deviation.
